# Biomechanical Effects of the MIND&GAIT Exercise Program on Sit-to-Stand and Marching in Place Motor Coordination in Institutionalized Older Adults: Implications for Functional Stability

**DOI:** 10.3390/healthcare14060770

**Published:** 2026-03-19

**Authors:** Cristiana Mercê, Susana Alfaiate, Fátima Ramalho, David Catela, Marco Branco

**Affiliations:** 1 Sport Sciences School of Rio Maior (ESDRM), Santarém Polytechnic University, Av. Dr. Mário Soares, 110, 2040-413 Rio Maior, Portugal; 090517001@esdrm.ipsantarem.pt (S.A.); fatimaramalho@esdrm.ipsantarem.pt (F.R.); catela@esdrm.ipsantarem.pt (D.C.); marcobranco@esdrm.ipsantarem.pt (M.B.); 2 Sport Physical Activity and Health Research & Innovation Center (SPRINT), Santarém Polytechnic University, Av. Dr. Mário Soares, 110, 2040-413 Rio Maior, Portugal; 3 Physical Activity and Health—Life Quality Research Centre (CIEQV), Santarém Polytechnic University, Complexo Andaluz, Apart 279, 2001-904 Santarém, Portugal; 4 Interdisciplinary Center for the Study of Human Performance (CIPER), Faculty of Human Kinetics, University of Lisbon, Cruz Quebrada-Dafundo, 1499-002 Lisboa, Portugal; 5 Quality Education—Life Quality Research Centre (CIEQV), Santarém Polytechnic University, Complexo Andaluz, Apart 279, 2001-904 Santarém, Portugal

**Keywords:** older adults, inertial sensors, non-linear dynamics, motor variability, functional mobility, motor development, exercise, group fitness, health, fall risk

## Abstract

**Highlights:**

**What are the main findings?**

- The MIND&GAIT program generated positive trends in motor variability and unpredictability in sit-to-stand and marching-in-place tasks, despite no statistically significant changes.
- Results suggest improvements in adaptive motor coordination, particularly during the marching-in-place task, with shifts toward more flexible and divergent movement patterns.

**What are the implications of the main findings?**

- These biomechanical trends suggest that MIND&GAIT may enhance functional stability and adaptability to perturbations, mechanisms essential to fall prevention.
- These exploratory results support the inclusion of structured exercise programs like MIND&GAIT as a valuable strategy for reducing fall vulnerability in institutionalized older adults.

**Abstract:**

**Background**: Motor decline associated with ageing compromises mobility, postural control and the ability, thereby increasing risk among older adults. Biomechanical characterization of movement, particularly using non-linear methods, offers a process-oriented approach capable of detecting subtle changes in motor coordination. The MIND&GAIT programme has previously demonstrated benefits in physical function in frail older individuals; however, its potential to improve motor coordination parameters that underpin fall risk remains insufficiently explored. **Objectives**: To analyse the impact of the MIND&GAIT program on motor coordination during sit-to-stand (STS) and walking tasks, two daily activities strongly associated with fall risk, using advanced non-linear and biomechanical metrics in institutionalized older adults. **Methods**: Fourteen institutionalized older adults (82.21 ± 7.14 years) participated. Three-dimensional acceleration and angular velocity were recorded using inertial sensors. Motor variability and predictability were quantified using the multivariate Lyapunov exponent (LyEM) and multivariate incremental entropy (MIE). STS (30 s) and walking-in-place (2 min) tasks were assessed pre- and post-intervention following a three-month, thrice-weekly programme. **Results**: Although no statistically significant differences emerged (*p*s > 0.05), trends were observed suggesting increases in LyEM during STS and in both MIE and LyEM during walking were found post-intervention. These exploratory findings may indicate enhanced motor complexity, stability and adaptability, features associated with reduced fall vulnerability. **Conclusions**: Despite the absence of statistical significance, the biomechanical trends observed suggest improvements in motor coordination patterns relevant to fall risk reduction in institutionalized older adults following the MIND&GAIT programme. These findings highlight the potential of structured exercise-based interventions for promoting safer movement behaviors in frail populations.

## 1. Introduction

Ageing affects the sensory systems involved in postural control, increasing whole-body instability and, in older adults, the likelihood of falls [1]. As sensory deficits accumulate with age, gait patterns also change, including greater variability and slower gait speed, which are associated with reduced ankle range of motion [2,3]. Gait is a gross motor task that requires balance, strength and coordination. For this reason, analyzing its spatio-temporal parameters, along with their variability, is essential for identifying pathologies and motor disorders [4,5]. The sit-to-stand movement is likewise a fundamental daily task, especially for older adults. It is crucial for maintaining independence, and numerous studies have demonstrated its association with lower-limb strength in older populations [1]. Similar to gait, difficulties in performing this movement can predict fall risk. These difficulties may arise from muscle weakness, balance impairments, joint limitations, or other physical constraints [6]. Physical inactivity is associated with poor motor control and reduced movement speed, whereas the practice of physical exercise can improve both functional fitness parameters and gait performance [7,8]. It therefore becomes pertinent to stimulate and assess not only the physical capacities of older adults, but also their coordinative abilities during everyday functional tasks.

In the fields of biomechanics and motor behavior research, several methodologies and instruments have been used [9]. Inertial Measurement Units (IMUs), stand out as key tools for movement analysis, providing three-dimensional data on angular velocity and linear acceleration. These small instruments, typically composed by triaxial magnetometers, accelerometers and gyroscopes, allow reliable quantification of functional tasks, such as balance assessments or impact transmission across body segments. IMUs are practical, easy to use, and support detailed examination of linear and angular movements, enabling the estimation of temporal, kinematic and dynamic parameters [9].

To achieve a deeper understanding of variability and stability patterns in movement, non-linear analytical measures have gained increasing relevance [10]. Entropy is a metric capable of quantifying and predicting irregularities in time series data, offering deeper and more complex insight into movement mechanisms. Non-linear analysis, particularly entropy, has proven to be an important tool for understanding the complexity of human movement. Unlike traditional linear analyses, which may fail to capture the richness of dynamic data, entropy provides a means to quantify irregularity and predictability in time series [11].

Multivariate Incremental Entropy (MIE) is highly effective for analyzing short signals or limited datasets, as it is particularly sensitive to subtle changes in the underlying structure, indicating higher or lower randomness and predictability in motor behavior [12]. Higher MIE values reflect greater unpredictability within the system, meaning lower predictability, which corresponds to an increased adaptive capacity when facing perturbations. Ageing is typically associated with a reduction in such adaptive capacities when responding to stability challenges [13,14]. Given that frail, institutionalized older adults commonly exhibit diminished postural control, slower compensatory reactions and higher susceptibility to balance disturbances, MIE becomes particularly relevant for capturing these subtle deficits [13]. By quantifying the degree of unpredictability in motor responses, MIE can differentiate between preserved and impaired adaptive capacity—information that traditional linear metrics often fail to detect in this population. Thus, MIE provides a sensitive framework to examine how ageing and frailty impact motor behavior dynamics and system stability, supporting the design of more targeted interventions to enhance adaptation in older populations.

The calculation of the Lyapunov exponent (LyE), also a non-linear metric, is widely recognized in dynamic systems analysis and is considered an efficient method for studying motor behavior because it allows the assessment of the variability and adaptability of a system when exposed to perturbations [15,16]. This technique has been used in several tests, including sit-to-stand, finger tapping, and gait tasks [11]. The computation of the maximum Lyapunov exponent enables the analysis of complex systems by examining whether system trajectories converge (i.e., negative values) or diverge (i.e., positive values), thereby assessing variability and behavioral stability. Higher LyE values reflect greater variability in behavior, as well as greater adaptive capacity when the system is exposed to perturbations [17]. Nevertheless, it is important, to distinguish adaptive variability, characterized by flexible and functional adjustments to perturbations, from pathological instability, which reflects excessive, uncontrolled divergence of system trajectories. While moderate increases in LyE suggest healthy adaptability, disproportionately high values may indicate compromised motor control and instability rather than functional responsiveness [18].

Bearing in mind that non-linear measures such as MIE and the LyE are sensitively capture the complexity, variability, and adaptive capacity of motor control [14,17]. And that within the ageing process the changes in fundamental tasks as gait and sit-to-stand can reflect functional instability and greater vulnerability to perturbations and falls [1]. It is pertinent to evaluate interventions that act directly on these coordination determinants. MIND&GAIT is a multicomponent program validated for older adults [19], designed to improve functional fitness and mobility in seniors by combining structured physical exercise (i.e., lower-limb strength, balance, coordination, and mobility) with progressive cognitive stimulation. Through tasks and progressions that demand efficient spatiotemporal organization of movement, postural adjustment, and adaptive responses to perturbations, the program inherently targets mechanisms associated with fall prevention. Nevertheless, despite the established relevance of non-linear metrics for understanding motor coordination, there is a clear research gap: no studies to date have examined whether MIND&GAIT produces measurable changes in non-linear motor coordination metrics, as MIE or LyE, in institutionalized older adults. This limits current understanding of how multicomponent training may influence system adaptability and stability at a dynamical level.

To address this gap, and following the need to characterize changes in motor coordination in ecologically valid tasks using inertial sensors (IMUs) and non-linear metrics, the present study aimed to analyze the effects of the MIND&GAIT exercise program on gross motor coordination, through variability (LyE) and predictability (Entropy), in the sit-to-stand (STS) and marching in place tasks.

## 2. Materials and Methods

### 2.1. Study Design

A quasi-experimental design with a single experimental group and a quantitative approach. The physical exercise program included 37 sessions in total, during 12 weeks with three sessions per week. The physical sessions were performed at light to moderate intensity. The protocol included three phases: (i) pre-intervention assessment, (ii) intervention, and (iii) post-intervention assessment.

This study was designed to prioritize a process-oriented analysis of motor coordination using nonlinear metrics (LyEM and MIE) during ecologically valid tasks (STS and marching-in-place). Assessments were conducted within one week before and after the 12-week intervention (37 sessions). Sample size was constrained by the single-center exploratory scope; no a priori power calculation was performed. Once all residents who met the eligibility criteria agreed to participate, it was not possible to form an equivalent control group. Deliberately excluding eligible participants would not have been ethically acceptable in this context. In this sense, a single-group quasi-experimental design was adopted. Without a control group, threats to internal validity (e.g., learning effects, day-to-day variability, health fluctuations, regression to the mean) cannot be excluded; we standardized assessor, setting, and sensor placement to mitigate procedural variance.

Ethical approval for this study was obtained from the Ethics Committee of the Polytechnic University of Santarém (approval number 10-2024 ESDRM).

Reporting followed the TREND statement for non-randomized evaluations and relevant STROBE items for observational reporting; a completed checklist is available upon request.

### 2.2. Sample

The program was implemented at an elderly day center located in a city in central Portugal. All residents who met the inclusion criteria were invited to participate, totaling an initial number of 21 older adults. Due to hospitalization (n = 1), change of institution (n = 4), death (n = 1), and worsening of pre-existing pathologies (n = 1), only 14 participants completed the program and its assessments. Therefore, this was a convenience sample.

The final sample was composed by 14 older adults of both sexes (10 women), aged between 70 and 93 years (M = 82.69 ± 6.81 years). Inclusion criteria included non-participation in other physical exercise programs, the absence of neurodegenerative diseases, and no contraindications for physical exercise [7].

### 2.3. MIND&GAIT Program

The MIND&GAIT programme, previously developed and validated for frail older adults [19], was adapted in the present study to the institutionalized elderly population. To optimize its applicability and ensure sufficient training stimulus in this context, the weekly frequency was increased from the original two to three sessions per week. The program included 37 sessions over 12 weeks with three sessions a week, each lasting 45 to 60 min.

The programme focused on functional capacity such as postural stability, lower-limb strength, balance, agility and aerobic endurance. All exercises were structured progressively and adjusted to each participant’s individual abilities. Sessions’ intensity was monitored using the Modified Borg Rating of Perceived Exertion, and targeting that the training remained within light (1–4) to moderate (5–6) effort levels.

The program included aerobic, balance, strength and flexibility exercises. It also included a more playful aspect to promote socializing and the well-being of the participants, while also emphasizing posture stability, strength, balance, agility and aerobic capacity. All exercises and games were performed with an intensity adjusted to each participant’s abilities.

In terms of its periodization, the program included an initial adaptation and learning phase, which sought to increase body awareness (i.e., acquisition of neutral alignment, pelvic and scapular alignment, control of spinal mobility), identify breathing patterns, and train posture and balance, training weight distribution across both feet. As the program progressed and in line with the participants’ progress, the intensity was gradually increased. The sessions lasted approximately 45 to 60 min and were divided into three parts: (i) warm-up, 5 to 10 min, including body awareness, breathing, joint warm-up; (ii) main phase: 25–35 min, aerobic, strength, and functional training including balance; (iii) cool down and stretching, 5–10 min [19]. The end of the sessions always included a game, a dance, coordination exercises or object manipulation. Table 1 below shows the structure of the classes.

All sessions were delivered by a certified exercise professional with specific training in the MIND&GAIT programme and prior experience delivering exercise sessions to frail and institutionalized older adults. At the time of implementation, the instructor was a Master’s student specializing in this field and had received dedicated training from the programme’s development team, ensuring fidelity to the original protocol and safe, correct execution of all exercises. All session plans and Borg RPE ranges were systematically logged to monitor fidelity.

### 2.4. Motor Coordination Assessment

To assess gross motor coordination in daily life representative tasks, the 2 min marching test and the sit-to-stand (STS) test were selected, both part of the functional battery proposed by Rikli & Jones [20] and validated for evaluating physical fitness in older adults. The 2 min marching test consisted of marching in place for 2 min, raising the knee to the midpoint between the patella and the iliac crest; this point was individually identified for each participant and visibly marked at the testing site to ensure uniform execution. The marching-in-place test was selected because it is a validated protocol for older adults within the Rikli and Jones [20] functional battery and offers greater safety and feasibility in frail, institutionalized populations. This dynamic task captures rhythmic and variability-related components of gait while ensuring a controlled environment and minimizing fall risk. Performing the task in place also enhances signal standardization required for non-linear analysis. Nevertheless, it is acknowledged that its ecological validity is lower than that of overground walking. The STS test consisted of standing up and sitting down on a chair as many times as possible within 30 s, with the arms crossed over the chest [20].

Three-dimensional linear acceleration and angular velocity data were collected using a small inertial sensor equipped with a gyroscope (MPU 9250, Invensense, TDK Corporation, San Jose, CA, USA) [11]. All tests were administered by the same evaluators and in the same location to ensure procedural consistency. For both tasks, the sensor was positioned 10 cm above the anatomical landmark tibiale laterale. This placement was chosen to minimize soft-tissue artefacts resulting from displacement of the skin and surrounding tissues during knee flexion and extension, while maintaining a stable bony reference [9,11].

Data acquisition was performed using a custom software application developed by the authors in Python 3.12.10 with an HTML-based graphical interface and executed on a Windows 11 operating system. The application was designed to receive and store inertial sensor data transmitted wirelessly via Wi-Fi from the IMU devices. Data were transmitted using the UDP protocol through a dedicated communication port and recorded in real time without any preprocessing or modification of the original signal. The application stored the timestamps generated by the sensors together with the transmitted inertial measurements, preserving the original temporal structure of the signal. The acquired data were saved as plain text files (.txt) and subsequently used for offline processing and analysis. Subsequent signal processing and nonlinear analyses were conducted in MATLAB R2025a [11,21].

The data collected by the sensors were transmitted via Wi-Fi to a laptop computer using dedicated software, then exported to an Excel file and processed using a custom routine developed in MATLAB [11,21]. In this routine, the signal sampling frequency was determined from the time vector as the inverse of the mean sampling interval and was: (i) visually inspected and plotted for manual trimming before and after task execution; (ii) the trimmed raw signal was preprocessed using an adaptive band-pass filtering procedure. For each axis independently, a Fast Fourier Transform (FFT) was computed to obtain the single-sided amplitude spectrum. Frequencies below 0.1 Hz and above the Nyquist frequency were excluded. Dominant frequency components were identified using a peak detection algorithm with a threshold set at 1% of the maximum spectral amplitude. Lower and upper cutoff frequencies were adaptively defined by expanding the minimum and maximum detected peak frequencies by ±1 Hz. The resulting passband was constrained within 0.1–5 Hz to ensure physiologically plausible movement frequencies. The signal was then filtered using a 4th-order Butterworth band-pass filter, with cutoff frequencies normalized to the Nyquist frequency, followed by a zero-phase forward–backward filtering to prevent phase distortion and preserve the temporal structure of the signal [22,23]; (iii) For each axis, local maxima and minima were identified using a finding peaks function, applying a minimum inter-peak distance of 0.5 s and a prominence threshold set at 20% of the signal range to reduce spurious detections, with visual confirmation of the time series for verification; (iv) the filtered triaxial inertial signal was transformed to absolute values, and no other normalizations were made to the signal; (v) Phase-space parameters were estimated as follows: the time delay (τ) was defined as the first lag where the autocorrelation dropped below 0.1 (up to a maximum lag of 500 samples), the embedding dimension (m) was obtained using an optimized false-nearest-neighbors approach with maximum dimension set to 4, and the tolerance parameter (R) was estimated via an optimized fraction-based procedure. The reconstruction of system dynamics from delay coordinates is based on embedding theory [24], with FNN used for practical embedding dimension estimation [25]. Final multivariate parameters were combined using the maximum τ and m across axes and the minimum R across axes. (vi) Multivariate Incremental Entropy was computed over sliding windows whose length and step were defined from the mean cycle period estimated from the detected peaks, ensuring windowing consistent with task periodicity; (vii) for each recorded trial, the multivariate largest Lyapunov exponent (LyEM) was estimated from the triaxial inertial signal (X, Y, Z) simultaneously, using a Wolf-based approach to quantify divergence of nearby trajectories in phase space and capture the dynamics’ sensitivity to initial conditions [26].

Approximately 30 min before testing, the sensors were calibrated using the IMUDataReceiver software Version 1.0, which performs the initial signal processing.

During data collection, participants initiated the movement a few seconds after sensor activation. The analyzed movement segments were extracted from the first acceleration peak, corresponding to the start of the task (first extension to stand up in the STS test or first step in the marching task), to the last acceleration peak recorded for each task.

Similarly, Multivariate Incremental Entropy (MIE) was calculated according to established procedures in the literature [12] and adjusted across all axes, thus representing the multivariate complexity and predictability of the signal.

### 2.5. Data Treatment

Statistical analysis was performed using the Statistical Package for the Social Sciences software (version 29, SPSS Science, Chicago, IL, USA). Descriptive statistics were calculated to characterize the sample and all variables under study. The Shapiro–Wilk normality test was applied. According to the normality of the variables, paired *t*-tests or Wilcoxon tests were used to examine significant changes in multivariate incremental entropy and multivariate Lyapunov measures in the marching in place and STS tasks between pre- and post-intervention assessments. Effect sizes were calculated to complement the non-parametric analyses. For the Wilcoxon signed-rank tests, effect size (r) was derived using the formula r = Z/√N, in line with recommended procedures for non-parametric paired comparisons [27]. Magnitude was interpreted according to Cohen’s benchmarks (small = 0.10, medium = 0.30, large = 0.50).

## 3. Results

The implementation of the program comprised 37 exercise sessions, and the participants’ adherence and retention were exceptionally high. The participants attended on average 35.96 ± 1.56 sessions. Among the fourteen older adults, eight attended all sessions, four attended 35 sessions, one attended 34 sessions, and one attended 32 sessions.

Variability (LyEM) and unpredictability (MIE), two variables that allow the assessment of motor behavior, were evaluated in both the sit-to-stand (STS) and marching-in-place (MIP) tests both pre- and post-intervention. No significant differences were observed (all *p*s > 0.05). Nevertheless, increases in mean values were noted for LyEM in the STS test, as well as for both MIE and LyEM in the gait test, as presented in Table 2.

To complement the non-significant inferential results, effect sizes (*r*) from the Wilcoxon tests were calculated. These results indicated small effects in the STS task (MIE: *r* = 0.084; LyEM: *r* = 0.142) and small-to-moderate effects in the marching-in-place task (MIE: *r* = 0.251; LyEM: *r* = 0.357), suggesting slight but non-negligible magnitudes of change.

## 4. Discussion

The present study aimed to evaluate the effect of the MIND&GAIT program on motor coordination during the sit-to-stand (STS) and 2 min walking (marching in place) tests. Different methodologies may be used to assess motor coordination, including more traditional approaches such as phase plot analysis [28,29], as well as more innovative approaches such as nonlinear methods. Among nonlinear methods, the study of variability through multivariate Lyapunov Exponents (LyEM) and predictability through Multivariate Incremental Entropy (MIE) are two of the most widely recommended tools [12,15,17,30]. Importantly, these nonlinear variables are particularly relevant in a fall-prevention framework once they provide information about movement adaptability and the capacity to respond to perturbations, two key determinants of functional stability in older adults [10,31]. Although the present study focused exclusively on process-oriented nonlinear metrics, these measures are conceptually linked to more product-oriented and traditional indicators of functional mobility. Greater movement variability and complexity, when organized adaptively, have been associated with better performance in tasks such as gait speed, transitional movements, and balance recovery [31,32]. In this sense, increases in LyEM or MIE may reflect underlying mechanisms that support functional outcomes like safer gait or more efficient sit-to-stand performance. While we did not analyze clinical performance indicators in this manuscript, these relationships will be explored in detail in a separate study using the linear outputs collected with the same protocol. In addition to improving older adults’ general health, physical exercise (PE) offers benefits for physical fitness, namely endurance, mobility, agility, speed, and motor coordination, a key factor for preventing the loss of functional capacities and dependency [33,34]. A lack of coordinative abilities leads to instability, which increases with aging and contributes to one of the major factors underlying loss of independence in older adults: falls. These events often result in physical and psychological impairments [35]. Gait stability depends on the individual’s ability to maintain balance in the presence of disturbances. In the MIP task, both nonlinear indicators increased from pre- to post-intervention, with small-to-moderate effect sizes. Exactly, MIE increased from 0.052 to 0.088 and LyEM from 0.039 to 0.307, suggesting a tendency toward greater movement complexity and dynamic variability in the lower-limb movement pattern. From a biomechanical perspective, rhythmic stepping requires continuous regulation of limb oscillation, foot placement, and the transition between support and swing phases to maintain dynamic balance. Because the inertial sensor was positioned above the tibiale laterale, the recorded signal primarily reflects the dynamics of the distal lower-limb segment during stepping. In this context, the observed increases in MIE and LyEM may indicate a greater capacity to adapt the temporal organization and variability of lower-limb motion, which may be compatible with more flexible coordinative control during locomotor-like activity. These effects were more evident in MIP than in STS, which is consistent with the greater rhythmic and balance-related demands of the stepping task.

Dynamic balance and lower-limb coordination are particularly important when responding to unexpected situations [36]. Structured and supervised PE can also enhance lower-limb muscle strength, playing an essential role in the maintenance and improvement of functional capacity [37]. Given that falls often occur during transitional movements (e.g., rising from a chair) and during walking when unexpected perturbations arise, examining STS and marching in place (MIP) through variability and predictability metrics may help to capture subtle changes in functional stability that are not always detected by conventional outcomes. From a biomechanical perspective, the STS task involves a coordinated transition from a stable seated posture to upright standing, requiring forward displacement of the center of mass followed by extension of the lower-limb joints. This movement requires precise temporal coordination between load transfer, ankle and knee extension, and stabilization during seat-off. Because of the location of the inertial sensor, the recorded signal primarily reflects the dynamics of the distal lower-limb segment during this transition. In this context, the nonlinear metrics may capture subtle changes in the temporal organization and variability of the lower-limb movement pattern during the load-transfer and extension phases of STS. The observed increase in LyEM together with the small variation observed in MIE may therefore be compatible with slight modifications in the coordinative control of the lower limb during the postural transition, potentially reflecting a movement pattern that preserves adaptability while maintaining an organized temporal structure of the task.

From this perspective, it would be expected that older adults participating in the MIND&GAIT program would show increases in variability and unpredictability. However, contrary to the hypotheses, no statistically significant differences were found in any variable or task under analysis. Despite the lack of statistical significance, the effect sizes provide additional insight into the magnitude of the observed changes. In the STS task, effect sizes were small (*r* = 0.084–0.142), whereas MIP presented small-to-moderate magnitudes (*r* = 0.251–0.357). These values reinforce the presence of subtle but directionally consistent improvements, particularly in MIP, which align with the expected sensitivity of non-linear metrics to capture early adaptations in motor coordination. Importantly, these effect-size patterns also indicate that the STS task did not exhibit comparable directional trends, especially regarding entropy, where mean MIE values decreased rather than increased. From a conceptual standpoint, the relationship between variability/entropy and functional performance is non-linear; consequently, increases in LyEM or MIE do not inherently denote “better” control. Depending on task demands, the amplitude and organization of fluctuations, greater variability can indicate either adaptive flexibility or excess noise/instability [38,39]. We therefore interpret the present changes as compatible with early adaptations in coordinative behavior predominantly in the MIP task, where increases were observed in both LyEM and MIE. In contrast, the STS task did not show a clear or consistent directional pattern, particularly for MIE, whose mean values decreased from pre- to post-intervention. Nevertheless, despite the lack of statistical significance, patterns of improvement in motor coordination can be observed. These improvements appear primarily in the MIP task, which showed increases in mean and maximum variability and unpredictability. For the STS task, only LyEM exhibited an increase in mean values, whereas MIE demonstrated a slight reduction, indicating that adaptations in this task were weaker or not directionally consistent. The Lyapunov Exponent quantifies a system’s ability to mitigate small perturbations, with higher values indicating faster divergence of nearby trajectories in the reconstructed state space, which may reflect increased sensitivity of the movement dynamics to small perturbations and potentially greater adaptability to changing task demands [10,17]. Within an exploratory fall-prevention framework, these increases are better interpreted as changes consistent with improved regulation when challenged with small destabilizing events, rather than as evidence of functional benefits (e.g., fall prevention) per se. At the same time, it should be acknowledged that “more variability” is not universally beneficial; in frail older adults, excessively high or poorly organized variability can reflect impaired control [39]. Therefore, the observed post-intervention increases are best interpreted as potentially reflecting greater adaptability, rather than instability, especially in the MIP task, which is accompanied by concurrent trends in entropy and changes in LyE sign (negative to positive) suggestive of more flexible dynamics [10,16,17].

Among older adults, this adaptive capacity tends to decline with aging, reducing interactions among system components, as well as overall variability and complexity [40]. Thus, the observed increase in mean LyE may be interpreted as an indicator of improved motor coordination. Another supporting indicator is the reduction in negative LyE values. In the pre-intervention assessment, negative LyE values were found in both tests, reflecting a convergent and less adaptable system [10,17]. After the intervention, a slight reduction in LyE negativity was found in the STS test, and a notable improvement in the MIP test, which exhibited a positive LyE, characteristic of more divergent and adaptable systems. From a clinical standpoint, a shift toward more adaptable (divergent) dynamics during MIP may be meaningful because the ability to face perturbations without settling into rigid patterns is closely linked to safer locomotion and reduced balance loss. In contrast, the STS task showed only minimal LyE changes, with no accompanying increase in entropy, suggesting weaker or less consistent adaptive reorganization.

Similarly, higher MIE values indicate greater complexity and uncertainty in the system being analyzed. This implies lower predictability and higher variability, suggesting a more dynamic and adaptable system [12,40]. During senescence, motor behavior, including MIP, becomes more regular and less adaptable [12]. Just as muscle tissues become more “rigid” with aging, coordinative abilities also deteriorate, typically resulting in lower MIE values. The high variability observed in certain nonlinear parameters likely reflects inter-individual heterogeneity within this frail institutionalized sample. Such variability is expected in cohorts with diverse functional profiles and may also indicate the influence of individual outliers. These factors should be considered when interpreting dispersion patterns in LyEM and MIE. Accordingly, the observed post-intervention trends toward higher MIE values may be viewed as compatible with enhanced coordinative flexibility, but only in the MIP task, while acknowledging that these findings are not accompanied by direct fall-related outcomes. In this context, greater movement complexity can be framed as a property that may support a wider repertoire of responses under perturbation; however, implications for fall prevention should be considered tentative and hypothesis-generating only. Therefore, the simultaneous upward trends in MIE and LyEM, despite non-significance, are best interpreted as preliminary indicators of more adaptable patterns, warranting confirmation in studies incorporating overground walking and fall-related endpoints. Taken together, any directional inferences from LyEM/MIE should be made with caution, as similar numerical changes can arise from distinct mechanisms (e.g., adaptive reorganization vs. increased signal noise). Future work should triangulate these nonlinear measures with overground walking, more clinical balance tests, and fall outcomes to determine whether the observed patterns reflect functionally meaningful adaptation.

It is important to note that, in addition to the small sample size, participants were institutionalized and highly frail, which limited the intensity of the exercise sessions. Completing only three sessions of predominantly low-intensity exercise per week may not have been sufficient to generate significant changes. The reduced sample size limits the analyses’ statistical power, increasing the likelihood of Type II error and may partially explain the absence of statistically significant results. Therefore, future studies are encouraged to adopt more regular weekly practice, ideally on a daily basis, to increase potential adaptations and benefits. Although marching-in-place is less ecologically valid than overground walking, its use provided a safe and standardized dynamic task compatible with non-linear analysis in this single-institution, frail cohort. Future studies should complement this approach with overground walking under controlled linear paths to enhance ecological validity while preserving analytical consistency.

Given the quasi-experimental pre–post design without a control group, the internal validity of the study is limited. Potential influences such as learning effects, natural day-to-day variability, fluctuations in health status or medication, and regression to the mean cannot be excluded. The absence of a control group limits the interpretation of the data, especially because no reference values for these tests and for the nonlinear (innovative) methodologies that were applied. It is recommended that future studies include two control groups: one composed of frail older adults who do not carry out the exercise program, as this group would make it possible to confirm that the changes, i.e., the possible improvements observed, are indeed the result of the program; and a second control group composed of non-frail and physically active older adults. Comparing the results and improvements observed in the frail group with those of non-frail, active older adults would make it possible to determine whether the changes approach the performance levels expected in healthier and more physically active older populations. Generalizability is limited to similar institutionalized, frail cohorts under supervised low-to-moderate-intensity programs. Future research should also explore the optimal duration and intensity of such exercise programs in order to maximize health and functional benefits for older adults.

## 5. Conclusions

This exploratory study did not detect statistically significant pre- to post-intervention changes in non-linear motor coordination metrics; however, small-to-moderate effect sizes, particularly during marching in place, suggest trends compatible with increased motor complexity and adaptability following participation in MIND&GAIT. These patterns should be interpreted as preliminary and hypothesis-generating, rather than as evidence of confirmed functional benefits. In line with the study design, sample size and frailty profile, no causal inferences can be drawn.

Regarding potential implications for stability and fall risk, the observed changes in LyEM and MIE may be consistent with mechanisms underpinning more flexible and adaptive coordination; nevertheless, such interpretations remain tentative, as no direct fall-related outcomes were assessed. Future research with larger samples, controlled overground walking protocols, appropriate control groups, and fall-relevant endpoints (e.g., prospective fall incidence or validated clinical proxies) is needed to verify whether these biomechanical trends translate into clinically meaningful improvements.

Taken together, the present findings highlight the feasibility of applying non-linear analyses with wearable sensors in institutionalized, frail older adults and motivate further investigation. Until corroborated by adequately powered trials, the effects observed here should be viewed as initial signals that warrant confirmation, rather than as definitive evidence of intervention efficacy.

## Figures and Tables

**Table 1:** Systematization of the class structure.

Phases	Scope	Aims	Content	Method
Warm up 5 to 10 min	Gradual transition from the pre-exercise phase (rest) to the exercise phase	Gradual warming up of muscles and joints Preparation for exercise	Body awareness Weight control through support Breathing General joint mobility	Light intensity Lower and upper limbs Local joint mobility General joint mobility Static stretching
Main Phase 30 to 35 min	Aerobic training	Improvement of cardiorespiratory capacity Coordination	Walking Coordination and sensorimotor exercises (visual and auditory stimuli) Rhythm Agility Laterality	Dynamic exercises HR ^1^—range of 40 to 50% of maximum heart rate Borg scale 3 to 6 (scale of 0 to 10)
	Strength training	Ability to produce tension in a specific muscle group Large muscle groups Work Muscle strength ADLs ^2^ Replication	Lower muscles/upper muscles Agonist/antagonist Flexor muscles/extensor muscles Core activation	5 to 8 exercises 8 to 12 repetitions 2 to 3 sets 50 to 60% of 1 RM ^3^ 2 min recovery
Cool down/Stretching 5 to 10 min	Transition between exercise and rest Flexibility Balance	Decreased heart and respiratory rates Muscle relaxation	Stretching, relaxation, and breathing Joint mobility	Calm music Interactive game Slow movements Static/dynamic stretching Comfortable joint movement, without causing pain

^1^ Heart rate, ^2^ activities of daily life, ^3^ one-repetition maximum.

**Table 2:** Presentation of the descriptive and inferential statistics for multivariate incremental entropy (MIE) and multivariate Lyapunov (LyEM) measures across the two tasks, sit-to-stand (STS) and gait, and for the pre- and post-intervention assessments.

Tasks	Variable_Assessment	M	SD	Med	Min	Max	Tendency	*p*	*r*
STS	MIE_Pre-	0.015	0.022	0.006	0.000	0.081	↓	0.753	0.084
	MIE_Post-	0.010	0.008	0.010	0.000	0.029			
	LyEM_Pre-	0.195	0.326	0.052	−0.003	1.123	↑	0.594	0.142
	LyEM_Post-	0.624	1.804	0.025	−0.009	6.785			
MIP	MIE_Pre-	0.052	0.058	0.021	0.000	0.158	↑	0.347	0.251
	MIE_Post-	0.088	0.092	0.087	0.000	0.284			
	LyEM_Pre-	0.039	0.083	0.016	−0.086	0.237	↑	0.182	0.357
	LyEM_Post-	0.307	0.678	0.035	0.012	2.362			

M—mean, SD—standard deviation, Med—median, Min—minimum, Max—maximum, Pre—pre-intervention, Post—post-intervention, ↓—decrease from pre- to post-intervention, ↑—increase from pre- to post-intervention.

## Data Availability

The data are not publicly available due to ethical restrictions. However, portions of the dataset may be made available upon reasonable request and subject to approval by the institutional ethics committee. The ethics committee only authorized data sharing among the researchers responsible for the study.

## Abbreviations

The following abbreviations are used in this manuscript:

ADLs	Activities of Daily Life
FFT	Fast Fourier Transform
FNN	False-Nearest-Neighbors
HR	Heart Rate
IMU/IMUs	Inertial Measurement Unit(s)
LyE	Lyapunov Exponent
LyEM	Multivariate Lyapunov Exponent
MIE	Multivariate Incremental Entropy
MIP	Marching in place
PE	Directory of open access journals
PE	Physical Exercise
RPE	Rating of Perceived Exertion (Modified Borg)
STROBE	Strengthening the Reporting of Observational Studies in Epidemiology
STS	Sit-to-Stand
TREND	Transparent Reporting of Evaluations with Nonrandomized Designs
1RM	One-Repetition Maximum
3D	Three-Dimensional
